# Prevalence and Characteristics of Patients with Obstructive Sleep Apnea and Chronic Obstructive Pulmonary Disease: Overlap Syndrome

**DOI:** 10.3390/life14050547

**Published:** 2024-04-25

**Authors:** Michail Fanaridis, Izolde Bouloukaki, Georgios Stathakis, Paschalis Steiropoulos, Nikos Tzanakis, Violeta Moniaki, Eleni Mavroudi, Ioanna Tsiligianni, Sophia Schiza

**Affiliations:** 1 Sleep Disorders Center, Department of Respiratory Medicine, School of Medicine, University of Crete, 70013 Heraklion, Greece; michfana@gmail.com (M.F.); stathakisgg@gmail.com (G.S.); vmoniaki@yahoo.gr (V.M.); elenima23@hotmail.com (E.M.); schizas@uoc.gr (S.S.); 2 Department of Social Medicine, School of Medicine, University of Crete, 71410 Heraklion, Greece; i.tsiligianni@uoc.gr; 3 Department of Respiratory Medicine, Medical School, Democritus University of Thrace, University General Hospital Dragana, 68100 Alexandroupolis, Greece; steiropoulos@yahoo.com; 4 Department of Thoracic Medicine, University Hospital of Heraklion, Medical School, University of Crete, 70013 Heraklion, Greece; tzanakisn@uoc.gr

**Keywords:** obstructive sleep apnea, chronic obstructive pulmonary disease, overlap syndrome, polysomnography, sleep architecture, cardiovascular disease

## Abstract

Overlap syndrome (OVS) is a distinct clinical entity that seems to result in potential cardiovascular consequences. We aimed to estimate the prevalence and risk factors for OVS in OSA patients and analyze clinical and PSG characteristics associated with OVS. In this cross-sectional study, 2616 patients evaluated for OSA underwent type-1 polysomnography (PSG). They were grouped as pure OSA (AHI > 15/h) and OVS patients. Demographics, PSG data, pulmonary function tests and arterial blood gases (ABGs) were compared between groups after adjustments for confounders. OSA was diagnosed in 2108 out of 2616 patients. Of those, 398 (19%) had OVS. Independent predictors of OVS were older age [OR: 5.386 (4.153–6.987)], current/former smoking [OR: 11.577 (7.232–18.532)], BMI [OR: 2.901 (2.082–4.044)] and ABG measurements [PaCO_2_ ≥ 45 OR: 4.648 (3.078–7.019), PO_2_ [OR: 0.934 (0.920–0.949)], HCO_3_^−^ [OR: 1.196 (1.133–1.263), all *p* < 0.001]. OVS was also associated with prevalent hypertension [OR: 1.345 (1.030–1.758), *p* = 0.03] and cardiovascular disease [OR: 1.617 (1.229–2.126), *p* < 0.001], depressive symptoms [OR: 1.741 (1.230–2.465), *p* = 0.002] and nocturia [OR: 1.944 (1.378–2.742), *p* < 0.001], as well as with indices of OSA severity. Disturbances in sleep architecture were more prominent in OVS expressed by lower %N3 and REM% and higher arousal index. Our data suggest that OVS is prevalent among OSA patients, with distinct clinical and PSG characteristics. These characteristics could be utilized as predictive factors for early identification and further evaluation of these patients towards desirable patient-reported outcomes.

## 1. Introduction

Chronic obstructive pulmonary disease (COPD) is a chronic respiratory disease with a significant global burden, currently ranking as the third leading cause of mortality worldwide, and its prevalence is on the rise [1]. The cost of COPD management is dependent on the morbidity of the disease, which can be influenced by other comorbidities such as cardiovascular diseases (CVDs) and obstructive sleep apnea (OSA) [2]. COPD frequently coexists with OSA, which has significant clinical implications for these patients, as OSA has been linked to several potential consequences, including excessive daytime sleepiness, impaired daytime functioning, metabolic dysfunction, and an increased risk of cardiovascular disease and mortality [3,4,5].

The overlap between COPD and OSA was initially described by David Flenley in 1985, who introduced the term overlap syndrome (OVS) [6]. While the term “overlap syndrome” can encompass the coexistence of OSA with various chronic respiratory diseases, such as asthma and idiopathic pulmonary fibrosis, it is primarily used to describe the association between OSA and COPD [5]. There is no consensus on the precise prevalence of OVS due to differences in the population under study, diagnostic approaches for OSA and COPD, and the limited awareness among physicians about OSA in COPD patients. Nevertheless, it is believed that roughly 10% of patients with one disease will also have the other disorder by chance [7,8,9]. In the general population, the prevalence of OVS falls within the range of 1% to 3.6%, while in COPD patients, it is higher, ranging from 3% to 66%. In patients with OSA, the prevalence falls between 7% and 55% [10,11,12,13].

While our understanding of its clinical and prognostic implications remains limited, emerging evidence indicates that patients with OVS may face worse clinical outcomes than those with either COPD or OSA alone [14,15]. Instead of being additive, the adverse effects of COPD and OSA may be intensified as a result of their synergistic relationship and, towards this, patients with OVS typically encounter more severe and prolonged nocturnal hypoxemia [16,17]. Additionally, there is evidence suggesting that patients with OVS experience worse clinical prognosis, characterized by poor quality of life, a higher likelihood of cardiovascular morbidity and mortality, hospitalization due to the acute exacerbation of COPD, and all-cause mortality, when compared to patients with COPD or OSA alone [9,18,19,20,21,22,23,24,25,26,27].

The aforementioned aspects highlight the importance of early identification and intervention for individuals with OVS. Therefore, the aim of our study was to estimate the prevalence and risk factors for OVS in a large cohort of Greek OSA patients and to analyze clinical and polysomnographic characteristics associated with this syndrome, which would be supportive for early patients’ identification, diagnosis, and individualized therapeutic plans towards desirable patient-reported outcomes.

## 2. Materials and Methods

### 2.1. Study Patients

We conducted a cross-sectional study for subjects evaluated for suspected OSA in the Sleep Disorders Center, Department of Respiratory Medicine, School of Medicine, University of Crete, in Greece during a 3-year period (2020–2023). The inclusion criteria were (1) age > 18 years (2) with moderate to severe OSA according to standard criteria and (3) an above-elementary school education. The exclusion criteria were refusal to participate, central sleep apnea syndromes, restrictive ventilator syndromes, severe congestive heart failure, a history of life-threatening arrhythmias, severe cardiomyopathy, long-term oxygen therapy, family or personal history of mental illness, drug or alcohol abuse, severe cognitive impairment, history of narcolepsy, or restless leg syndrome. All participants provided written informed consent, and ethical approval was provided by the University Hospital Ethics Committee (approval number: 16289/24-06-2020).

### 2.2. Data Collection

All patients underwent a detailed evaluation that included anthropometric parameters, including body mass index (BMI); medical and sleep history; associated conditions and comorbidities, including physician-based diagnosis for depression; smoking history; and alcohol intake. In addition, we performed pulmonary function tests (PFTs) and overnight attended polysomnography (PSG). Subjective daytime sleepiness, reflected by the Epworth Sleepiness Scale (ESS), insomnia reflected by the Athens Insomnia Scale, the quality of life reflected by the Short-Form-36 (SF-36) questionnaire, and the patient’s level of depression reflected by the Beck Depression Inventory (BDI) were recorded. The COPD Assessment Test (CAT) was utilized to assess the health status of patients with COPD.

### 2.3. Pulmonary Function Testing

PFTs were offered in all as subjects as part of the initial workup for OSA. These tests were performed by trained technicians following standardized procedures [28]. A post-bronchodilator fixed FEV_1_/FVC ratio < 70% was used to define the presence of persistent airflow limitation, indicating the presence of COPD, based on the Global Initiative for Chronic Obstructive Lung Disease (GOLD) criteria [29].

### 2.4. Questionnaires

#### 2.4.1. Epworth Sleepiness Scale

Currently, the ESS is the most commonly utilized self-reported assessment for measuring daytime sleepiness in clinical settings. The questionnaire is a straightforward, self-administered assessment consisting of eight items. Its purpose is to measure the likelihood of falling asleep in eight commonly encountered situations. A score of 10 or above indicates the presence of excessive daytime sleepiness [30].

#### 2.4.2. Beck Depression Inventory (BDI)

This 21-item questionnaire is a commonly used and highly reliable self-assessment tool for measuring depressive symptoms. The BDI quantifies the extent of depressive symptoms observed within the week prior. Each item requires the respondent to choose one or more options, rating them on a scale of 0 (no symptoms) to 3 (most severe level). The total scores, ranging from 0 to 63, are the sum of the highest level endorsed on each item. Scores that fall below 10 are considered to be in the normal range [31]. 

#### 2.4.3. SF-36

This questionnaire, consisting of 36 items, is a reliable and validated instrument for evaluating overall health (physical and mental) and quality of life. There are 8 domains within the SF-36, and each domain is individually scored on a scale of 0 (poorest) to 100 (best). The summary of the SF-36 scales can be divided into two dimensions: physical health and mental health. The scale ranges from 0 to 100, indicating the level of quality of life, with 100 being the best and 0 being the worst [32].

#### 2.4.4. COPD Assessment Test

The COPD Assessment Test (CAT) is a simple-to-complete questionnaire that assesses the self-reported impact of COPD on health status [33]. The CAT consists of eight items (cough, phlegm, chest tightness, breathlessness, limited activities, confidence in leaving home, sleeplessness, and energy), which the patient rates on a scale of 0 to 5. The score ranges from 0 to 40, with higher values indicating poorer health status. A cutoff point of 10 or above is used to determine the presence of poor health status.

### 2.5. Polysomnography

All patients underwent a single-night full diagnostic PSG study (Alice 5 Diagnostics System; Respironics, Murrysville, PA, USA) according to standard techniques, with monitoring of the electroencephalogram (using 3 electroencephalogram derivations: frontal, central, and occipital), electro-oculogram, electromyogram, flow (by oronasal thermistor and nasal air pressure transducer), thoracic and abdominal respiratory effort (by respiratory inductance plethysmography), pulse oximetry (SpO2), and body position monitoring. Snoring was recorded by a microphone placed on the anterior neck. The definition of apnea and hypopnea followed the American Academy of Sleep Medicine standard criteria [34]. The following parameters were analyzed: total sleep time (TST), sleep efficiency [SE (%)], wake after sleep onset (WASO), arousal index (AI), apnea–hypopnea index (AHI), oxygen desaturation index (ODI), resting room air pulse oximetry (SpO_2_), and sleep time with oxygen saturation < 90% (TST90) in minutes. The AHI, calculated as the number of apnea and hypopnea events per hour of sleep, was used to diagnose OSA and assess its severity. OSA was considered mild at AHI of 5 to <15 events/h, moderate at AHI of 15 to <30 events/h, and severe at AHI ≥ 30 events/h. In our study, only we included patients with an AHI ≥ 15/h.

### 2.6. Statistical Analysis

The results are presented as means ± SD for continuous variables if normally distributed and as medians (25th–75th percentile) if not. Qualitative variables are presented as absolute numbers (percentage). For comparisons between groups, a two-tailed *t* test for independent samples (for normally distributed data) or a Mann–Whitney U test (for non-normally distributed data) was utilized for continuous variables and the chi-square test for categorical variables. Factors associated with OVS were analyzed with bivariate logistic regression after adjustment for various potential explanatory variables, including age, sex, BMI, smoking status, indices of OSA severity, and comorbidities. For the purpose of this analysis, the term cardiovascular disease (CVD) referred to any of the following diseases: coronary disease and/or atrial fibrillation and/or Cerebro-Vascular Accident/Transient Ischemic Attack (CVA/TIA) and/or heart failure. Age was considered continuously and categorically as age groups of 18–59 and >60 years; BMI was also considered continuously and categorically as BMI groups of <30 and ≥30 kg/m^2^. Results were considered significant when *p* values were <0.05. Data were analyzed using SPSS software (version 25, SPSS Inc., Chicago, IL, USA).

## 3. Results

### 3.1. Clinical and PSG Characteristics of OVS Compared to Pure OSA Patients

**Figure 1:**
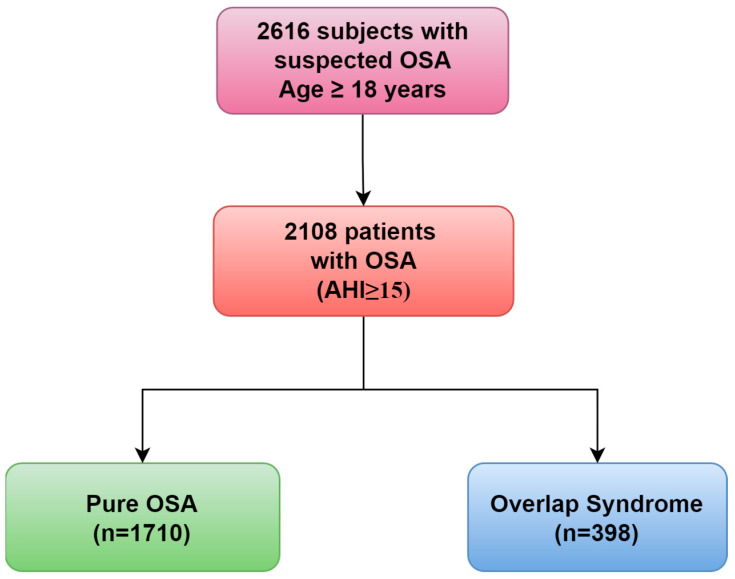
The flowchart of patients that were finally included.

Out of the 2616 adults who were assessed for suspected OSA, 2108 of them (81%) received a confirmed diagnosis of OSA. Among these, 1710 patients (81%) were classified as pure OSA, while 398 (19%) were classified as OVS (Figure 1). 

The patients’ clinical characteristics are outlined in Table 1. Patients diagnosed with OVS were notably older and had a higher obesity rate with larger neck circumferences, and they were mostly current or former smokers (all *p*-values less than 0.001). 

**Figure 2:**
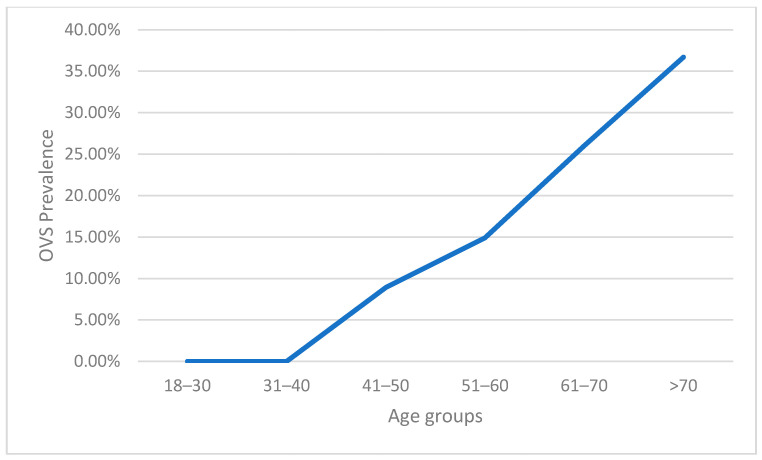
Prevalence of OVS across different age groups.

Interestingly, the prevalence of OVS increased across different age groups in individuals ≥ 40 years of age (Figure 2).

In the whole sample, the prevalence of co-morbidities ranged from 2% for CVA/TIA to 52% for hypertension. Variances were observed in the presentation of co-morbidities between the two groups. OVS patients exhibited a statistically significant higher prevalence of arterial hypertension, type 2 diabetes, coronary heart disease, atrial fibrillation, cardiac failure, CVA/TIA, and cancer in comparison to OSA patients. 

The study population’s PFT measurements and ABG analysis results are displayed in Table 2. The median FEV_1_/FVC ratio of OVS patients, as defined, was 63, while pure OSA patients had a significantly higher ratio of 81 (*p* < 0.001). The median FEV_1_ (% predicted) also differed significantly between OVS patients (66) and pure OSA patients (91) (*p* < 0.001). In addition, patients with OVS demonstrated significantly lower PaO_2_ levels, higher PaCO_2_ levels, and increased HCO_3_^−^ levels and a higher percentage of chronic hypercapnia (PaCO_2_ > 45 mmHg) (all *p* < 0.001) compared to pure OSA patients.

**Figure 3:**
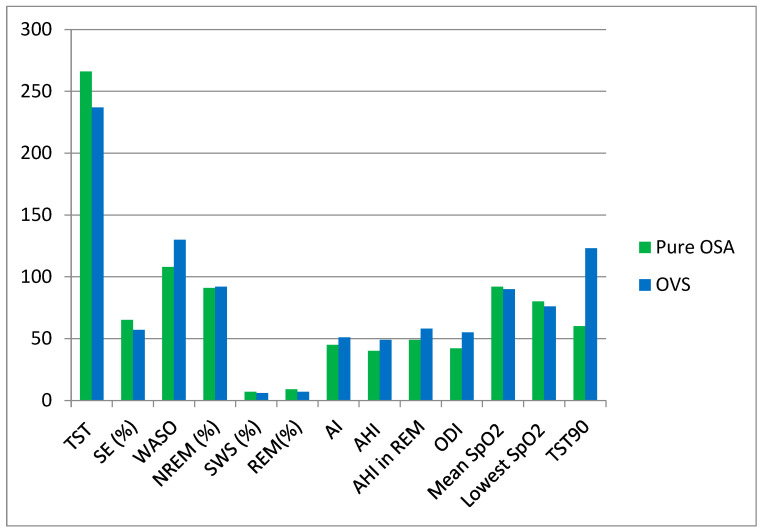
Differences in PSG characteristics between groups. NREM: Non-rapid eye movement; PSG: polysomnography; REM: rapid eye movement; SWS: Slow-wave sleep; TST: total sleep time; SE (%): sleep efficiency; WASO: wake after sleep onset; AI: arousal index; AHI: apnea–hypopnea index; ODI: oxygen desaturation index; SpO_2_: resting room air pulse oximetry; TST90: sleep time with oxygen saturation < 90%.

Figure 3 shows group differences in terms of PSG characteristics. Indices of OSA severity were worse in patients with OVS (medians AHI: 49 vs. 40, ODI: 55 vs. 42, SaO_2_: 90 vs. 92, minimum SaO_2_ 76 vs. 80, TST90: 123 vs. 60, all *p* < 0.001). Additionally, disturbances in sleep architecture were more prominent in patients with OVS, as expressed by lower TST, SE (%), %N3, and REM% values and higher WASO, NREM (%), and arousal index values (all *p* < 0.001).

As shown in Table 3, there was no difference in snoring, witnessed apneas, frequent awakenings, morning headaches, sleepiness, and insomnia symptoms between the groups. However, patients with OVS showed more severe functional impairments, which reached statistical significance in the Physical Functioning, Role Physical Bodily Pain, and Mental Health components of the SF-36 questionnaire. Furthermore, nocturia and depressive symptoms were more commonly observed in the OVS group. Importantly, nocturia was independently associated with indices of OSA severity [AHI, OR: 1.010 (1.006–1.015), ODI, OR: 1.012 (1.008–1.017), mean SaO_2_, OR: 0.856 (0.815–0.899), lowest SaO_2_ OR: 0.947 (0.931–0.963), TST90, OR: 1.005 (1.004–1.007), all *p* < 0.001)] and parameters of sleep architecture, including arousal index [OR: 1.016 (1.007–1.024), *p* < 0.001] and sleep efficiency [OR: 0.990 (0.981–0.999), *p* = 0.025], after adjustment for age, gender, BMI, and comorbidities including benign prostate hyperplasia. Furthermore, insomnia symptoms (AIS ≥ 6) [OR:1.012 (1.000–1.024), *p* = 0.048] were also associated with arousal index, and excessive daytime sleepiness (ESS ≥ 11) was associated with arousal index [OR: 1.018 (1.011–1.026), *p* < 0.001] and WASO [OR: 1.004 (1.002–1.006), *p* < 0.001]. 

### 3.2. Factors Associated with OVS in the Study Population

The results of the multivariable logistic regression analysis, specifically highlighting factors associated with OVS, are presented in Table 4. Independent predictors of OVS were older age, current/former smoking, truncal obesity, NC, waist/hip circumference ratio, and ABG measurements (PaCO_2_ ≥ 45, PO_2_, HCO_3_^−^).

The association between OVS and prevalent hypertension and CVD persisted even after adjusting for confounders. The same applied to depressive symptoms and nocturia as well as to the Physical Functioning, Role Physical Bodily Pain, and Mental Health components of the SF-36 questionnaire and most of indices of OSA severity (except AHI) and sleep architecture parameters (except NREM sleep). The association between physician diagnosis of depression and OVS was close to being statistically significant.

## 4. Discussion

In this study, we evaluated the prevalence, clinical characteristics and symptoms, and associated comorbidities of patients with OVS compared to patients with pure OSA. OVS was found to be present in 19% of patients who were 40 years old and older. Compared to the pure OSA group, patients in the OVS group were older, current or former smokers, with higher truncal obesity, and with worse arterial blood gas measurements and indices of OSA severity. Furthermore, patients in the OVS group had higher rates of comorbidities such as hypertension and cardiovascular disease and were more likely to experience depressive symptoms and nocturia, even after adjusting for confounders.

Various epidemiological studies have evaluated the prevalence of OVS in the OSA population, yielding estimates ranging from 7% to 55% [10,11,12,13]. We found a prevalence of 19% OVS among patients with OSA, similar to previous studies [9,11,35,36,37,38]. Prevalence rates exceeding 40% were found in three studies [39,40,41]; however, these studies had relatively small sample sizes and, in the majority of them, there were no accurate and objective measurements for OVS. 

It is widely accepted in most studies that patients diagnosed with OVS are notably older when compared to those with pure OSA [13,38,42,43,44]. There is evidence to suggest that patients with COPD are prone to developing sleep apnea as they age [17]. Furthermore, those with the COPD “blue bloater” phenotype, often accompanied by obesity, have a higher likelihood of developing sleep apnea [17]. Indeed, patients with COPD with a high BMI have a higher risk of OSA coincidence and, consequently, the development of OVS [44]. Notably, it was estimated that for each additional 1 kg/m^2^ in BMI, the risk of occurrence of OSA in COPD increases 2.552-fold [45]. In our study, patients in the OVS group exhibited significantly higher BMIs, neck circumferences, and waist/hip circumferences, which are indicative of truncal obesity, in contrast to individuals with pure OSA. In contrast, three recent studies showed that the OVS group had significantly lower BMIs and showed no significant difference in neck circumference than those with OSA alone [38,42,43]; however, one of these studies was conducted in the general population using only a screening questionnaire for OSA diagnosis [42], and in the others, the sample of OVS was relatively small, again raising the issue of accurate and objective OVS diagnosis [38,43].

In terms of clinical symptoms, individuals with OVS displayed distinct clinical characteristics compared with those with OSA alone. Specifically, OVS patients had a higher likelihood of experiencing nocturia and depressive symptoms compared to those with pure OSA. The frequency of nocturia was also higher in a large study comparing patients with OVS and OSA alone [9]. Patients with OVS may feel an increased awareness of bladder fullness and the need to urinate more often, secondary to increased frequency of awakenings [46]. Additionally, apneic episodes result in negative intrathoracic pressure and positive intra-abdominal pressure, which is then transmitted to the bladder. Notably, intermittent hypoxia can induce oxidative stress, also causing functional changes in the bladder of rats [47]. These changes are characterized by increased spontaneous bladder contractions, noncompliance, and detrusor instability, contributing to local alterations in the bladder, unrelated to urine production.

It is also worth noting that while 54% of the OVS population experienced symptoms of depression, only 15% received a medical diagnosis and treatment for depression. Moreover, the association between physician diagnosis of depression and OVS was close to being statistically significant, possibly due to the low number of patients diagnosed. Given that depression frequently coexists as a comorbidity in individuals diagnosed with COPD or OSA [48,49], it is reasonable to assume that those with OVS may be more susceptible to developing depression. However, there is a lack of comprehensive research on the frequency of depressive symptoms in the OVS population, and most of the existing studies have only included a small sample size of OVS patients. Given that OVS seems to impact different aspects of quality of life in our study, it is plausible that it can lead to restrictions in physical and social activities, ultimately resulting in emotional consequences such as depressive symptoms. Additionally, being exposed to disruptions in both macro and micro sleep patterns, as well as experiencing severe nocturnal hypoxemia and/or hypercapnia, could also be factors in the development of depression [50,51]. Available data indicate a higher prevalence of depression in OVS patients compared to those with pure COPD [52,53] but a similar prevalence compared to patients with OSA alone [9,42,54]. On the other hand, CPAP treatment has proven to be effective in reducing depression and enhancing the quality of life in individuals with OVS and OSA [55,56]. Therefore, it is crucial to promptly identify depression and provide appropriate treatment for patients with OVS, considering the potential negative impact of unrecognized and untreated depression on them, which can lead to increased fatigue and the greater utilization of healthcare resources [57,58,59].

This study also found that OVS displayed worsened indices of OSA severity, apart from AHI, when compared to pure OSA. These findings broadly support the work of other studies showing similar AHI levels in OSA patients with or without COPD [13,60,61] but sustained desaturation episodes [62] that can be more severe in patients with OVS than in patients with either COPD or OSA [13]. Additionally, disturbances in sleep architecture were more prominent in OVS, as expressed by lower SE (%), SWS (%TST), and REM (%TST) values and higher WASO and arousal index values. Previous research, which mostly involved a smaller number of participants, yielded conflicting results, as our findings are consistent with certain studies [13,63] but not with others [60,64,65]. Nevertheless, it is noteworthy that polysomnographic sleep quality parameters, mainly the arousal index, correlated with subjective clinical data, including nocturia, insomnia symptoms, and excessive daytime sleepiness.

Another important finding from our study is that patients in the OVS group were more likely to have comorbidities like hypertension and cardiovascular disease. This finding aligns with prior research, which suggests that OVS patients experience a higher burden of comorbidities, particularly cardiovascular ones, compared to those with only OSA or COPD [55]. While the impact of OVS on cardiovascular outcomes varied across different studies and remains uncertain [9,16,38,42,43,53,61,62,66,67,68,69,70,71,72,73,74,75,76,77,78], our study found that patients in the OVS group were linked to a 1.3-fold higher risk of developing hypertension and cardiovascular disease. Undoubtedly, the combined presence of COPD and OSA exerts a synergistic detrimental impact on cardiovascular health. This is primarily attributed to the interplay of nocturnal hypoxemia, systemic inflammation, diminished ventilatory drive, heightened expiratory airflow resistances, and alterations in pulmonary hemodynamics, all of which significantly contribute to CVD [13,76]. Our findings suggest that individuals with OVS experience breathing abnormalities both during wakefulness and sleep, which could partially explain the higher occurrence of CVD and arterial hypertension. Specifically, patients with OVS showed impairments in both daytime PaO_2_ and PCO_2_ levels, along with further decreases in oxygen saturation during sleep when compared to pure OSA patients, in line with previous studies [9,37,43,79,80]. Additionally, as expected, pulmonary function tests showed a significant decrease in patients with OVS due to the presence of COPD. A growing body of evidence highlights the importance of impaired lung function and hypoxia, especially during sleep, as major risk factors for the development of CVD [4,9,16,17,71,75,76,81,82]. OVS seems to be linked also with increased systemic inflammation which also may contribute to increased CVD [15]. This is evidenced by elevated levels of interleukin 6 (IL-6), high-sensitivity C-reactive protein (hs-CRP), and granulocyte colony-stimulating factor (G-CSF) in comparison to both healthy controls and individuals with OSA [15,78]; however, more research is needed to ascertain the potential synergistic effects of OSA and COPD on cardiovascular risk.

Our findings hold crucial implications for clinical practice. First, our research indicates a high prevalence of OVS in OSA patients, accompanied by distinct clinical and PSG characteristics. While age and smoking are consistently identified as predictors of COPD in various studies in the general population [83,84,85], other factors such as obesity, abnormal ABG measurements not aligned with OSA severity, nocturia, and depressive symptoms may also contribute to the prediction of COPD specifically in OSA populations. Clinicians may use these readily available characteristics as predictive factors for identifying COPD in OSA. These characteristics could improve their ability to diagnose early, create personalized treatment plans, and achieve desirable PROMs for patients with OVS. Furthermore, our findings suggest that the early diagnosis and proper treatment of OVS can potentially lower CV risk and increase survival rates in this patient population. 

The major strengths of our study are the large number of patients, detailed anthropometric and clinical evaluation and the objective diagnosis of OSA and OVS with the use of overnight PSG. However, it is important to acknowledge certain limitations of the current study. Firstly, patients included in the study were chosen via clinical referrals to our sleep center, which may have impacted the generalizability of our results to other populations. Secondly, due to the cross-sectional design of our study, we were unable to make any causal inferences. Thirdly, we did not record long-term sleep diaries which may have included subjective sleep duration and habits, both of which could have potentially influenced clinical symptoms. Future longitudinal studies may need to confirm the findings of this cross-sectional analysis.

## 5. Conclusions

Our data suggest that OVS is prevalent among patients with OSA, with distinct characteristics, a more severe OSA disease spectrum, and a higher degree of comorbidities. These findings build upon prior knowledge in the field and highlight the importance of early identification of the disease. Therefore, healthcare professionals should assess patients with COPD for OSA and vice versa in order to improve their prognosis and quality of life. Additional research is warranted to better understand the clinical significance, synergetic effect, and outcomes of OVS.

## Figures and Tables

**Table 1:** Characteristics of the study population.

	Total Population	OSA (AHI ≥ 15) (*n* = 2108)		*p* Value
	(*n* = 2108)	Pure OSA (*n* = 1710)	OVS (*n* = 398)	
**Demographics**				
Males	1625 (77%)	1289 (75%)	336 (84%)	<0.001
Age	57 ± 13	55 ± 13	66 ± 11	<0.001
Age ≥ 60 years	969 (46%)	675 (40%)	294 (74%)	<0.001
BMI	35 ± 7	35 ± 7	37 ± 7	<0.001
BMI ≥ 30	1581 (76%)	1241 (73%)	340 (87%)	<0.001
Waist/hip	1.0 ± 0.06	0.99 ± 0.06	1.02 ± 0.07	<0.001
Neck circumference	43 ± 5	43 ± 5	45 ± 5	<0.001
**Smoking Status**				
Never smoker	661 (31%)	638 (37%)	23 (6%)	<0.001
Current smoker	602 (29%)	460 (27%)	142 (36%)	
Former smoker	845 (40%)	612 (36%)	233 (59%)	
**Co-morbidities**				
Hypertension	1089 (52%)	820 (48%)	269 (68%)	<0.001
Coronary heart disease	247 (12%)	163 (10%)	84 (21%)	<0.001
Atrial fibrillation	138 (7%)	102 (6%)	36 (9%)	0.024
Cardiac failure	119 (6%)	67 (4%)	52 (13%)	<0.001
CVA/TIA	49 (2%)	29 (2%)	20 (5%)	<0.001
Cardiovascular disease	445 (21%)	302 (18%)	143 (36%)	<0.001
Diabetes type II	449 (21%)	324 (19%)	125 (32%)	<0.001
Cancer	92 (4%)	64 (4%)	28 (7%)	0.004
GERD	17 (13%)	13 (12%)	4 (15%)	0.66
Depression (on medication)	240 (11%)	185 (11%)	55 (14%)	0.08
Dyslipidemia	910 (43%)	728 (43%)	182 (46%)	0.24
Hypothyroidism	319 (15%)	262 (15%)	57 (15%)	0.62

BMI: body mass index; CVA/TIA: Cerebro-Vascular Accident/Transient Ischemic Attack; GERD: gastroesophageal reflux disease.

**Table 2:** Pulmonary function test measurements and ABG analysis results of the study population.

	Total Population	OSA (AHI ≥ 15) (*n* = 2108)		*p* Value
	(*n* = 2108)	Pure OSA (*n* = 1710)	OVS (*n* = 398)	
**PFTs**				
FEV_1_, % predicted	87 (73, 98)	91 (81, 101)	66 (53, 78)	<0.001
FVC, % predicted	86 (76, 99)	90 (81, 100)	71 (59, 83)	<0.001
FEV_1_/FVC	80 (76, 86)	81 (77, 87)	63 (59, 67)	<0.001
**ABG**				
pH	7.41 ± 0.04	7.41 ± 0.04	7.41 ± 0.03	0.20
PCO_2_ (mmHg)	40 ± 13	40 ± 15	44 ± 8	<0.001
PaCO_2_ ≥ 45 mmHg, *n* (%)	358 (17%)	171 (10%)	139 (35%)	<0.001
PO_2_ (mmHg)	78 ± 13	81 ± 12	70 ± 13	<0.001
HCO_3_^−^ (mmol/L)	26 ± 3	25 ± 3	27 ± 4	<0.001
SaO_2_ (%)	95 ± 5	96 ± 5	93 ± 4	<0.001

PFTs: pulmonary function tests; ABG: arterial blood gases; FEV_1_: forced expiratory volume in 1 s; FVC: forced vital capacity.

**Table 3:** Symptoms and clinical presentation of OVS compared to pure OSA.

	Total Population	OSA (AHI ≥ 15) (*n* = 2108)		*p* Value
	(*n* = 2108)	Pure OSA (*n* = 1710)	OVS (*n* = 398)	
**Nocturnal symptoms**				
Snoring	2065 (98%)	1676 (98%)	390 (98%)	0.613
Witnessed apneas	2044 (97%)	1658 (97%)	386 (97%)	0.744
AIS Score	8 ± 5	8 ± 5	9 ± 6	0.076
AIS ≥ 6 (%)	1433 (68%)	1162 (68%)	366 (67%)	0.779
Frequent awakenings	1686 (80%)	1385 (81%)	334 (84%)	0.079
Nocturia	1644 (78%)	1299 (76%)	346 (87%)	<0.001
**Diurnal symptoms**				
ESS score	11 ± 5	11 ± 6	11 ± 5	0.132
ESS ≥ 11	1180 (56%)	940 (55%)	234 (60%)	0.08
Morning headache	421 (20%)	342 (20%)	87 (22%)	0.513
Sleepiness at the wheel	442 (21%)	359 (21%)	91 (23%)	0.395
BDI score	8 (4, 14)	8 (4, 13)	10 (6, 16)	<0.001
BDI ≥ 10	927 (44%)	718 (42%)	231 (54%)	0.002
**COPD health status**				
CAT score		-	13 ± 7	
CAT score ≥ 10		-	278 (70%)	
**SF-36**				
PF	65 ± 26	67 ± 25	46 ± 27	0.008
RP	75 (25, 100)	75 (25, 100)	25 (0, 94))	0.004
BP	78 (55, 100)	80 (55, 100)	55 (23, 89)	0.001
GH	56 ± 21	56 ± 21	52 ± 24	0.515
VT	49 ± 24	49 ± 23	47 ± 24	0.798
SF	75 (50, 100)	75 (50, 100)	82 (28, 100)	0.565
RE	61 ± 20	67 ± 42	54 ± 48	0.355
MH	100 (0, 100)	100 (33, 100)	67 (0, 100)	0.018

AIS: Athens Insomnia Scale; BDI: Beck Depression Inventory; BP: bodily pain; ESS: Epworth Sleepiness Scale; GH: general health; MH: mental health; PF: physical functioning; RE: role emotional; RP: role physical; SF: social functioning; SF-36: Short-Form-36; VT: vitality.

**Table 4:** Multivariate logistic regression analysis of factors associated with OVS.

Variables	Adjusted OR (95% CI)	*p*-Value
**Sociodemographic factors**		
Males vs. females	1.405 (0.971–2.033)	0.071
Age, years	1.093 (1.080–1.107)	<0.001
Age group ≥ 60 years	5.386 (4.153–6.987)	<0.001
Current/former vs. no smoking	11.577 (7.232–18.532)	<0.001
Body mass index ≥ 30	2.901 (2.082–4.044)	<0.001
Neck circumference (cm)	1.128 (1.092–1.165)	<0.001
Waist/hip circumference ratio	120.923 (14.211–1028.937)	<0.001
**Health status factors**		
Hypertension	1.345 (1.030–1.758)	0.030
Cardiovascular disease	1.617 (1.229–2.126)	<0.001
Coronary heart disease	1.537 (1.124–2.103)	0.007
Atrial fibrillation	1.061 (0.694–1.624)	0.784
Cardiac failure	2.730 (1.811–4.116)	<0.001
CVA/TIA	2.026 (1.084–3.787)	0.027
Diabetes type 2	1.304 (0.989–1.720)	0.060
Cancer	1.626 (0.954–2.772)	0.074
Depression (on medications)	1.426 (1.001–2.031)	0.049
**Clinical symptoms**		
BDI ≥ 10	1.741 (1.230–2.465)	0.002
Athens Insomnia Scale Score ≥ 6	0.987 (0.613–1.589)	0.957
ESS ≥ 11	1.204 (0.936–1.549)	0.148
Nocturia	1.944 (1.378–2.742)	<0.001
**SF-36**		
PF	0.963 (0.936–0.989)	0.007
RP	0.987 (0.971–1.002)	0.036
BP	0.978 (0.953–1.004)	0.027
GH	0.983 (0.952–1.015)	0.292
VT	0.994 (0.968–1.020)	0.636
SF	1.007 (0.981–1.033)	0.623
RE	0.995 (0.981–1.009)	0.456
MH	1.008 (0.979–1.038)	0.031
**ABGs**		
PaCO_2_ ≥ 45 (mmHg)	4.648 (3.078–7.019)	<0.001
PO_2_ (mmHg)	0.934 (0.920–0.949)	<0.001
HCO_3_^−^ (mmol/L)	1.196 (1.133–1.263)	<0.001
**PSG characteristics**		
AHI	1.004 (0.999–1.008)	0.145
AHI in REM	1.010 (1.004–1.015)	<0.001
ODI	1.009 (1.004–1.014)	<0.001
Mean SpO_2_	0.823 (0.788–0.859)	<0.001
Minimum SpO_2_	0.942 (0.927–0.957)	<0.001
TST90	1.006 (1.005–1.008)	<0.001
Arousal Index	1.016 (1.007–1.026)	<0.001
Sleep efficiency (%)	0.979 (0.969–0.990)	<0.001
Wake after sleep onset (min)	1.005 (1.002 1–0.008)	<0.001
NREM (%TST)	1.000 (0.997–1.002)	0.970
SWS (%TST)	0.940 (0.883–1.002)	0.038
REM (%TST)	0.947 (0.901–0.996)	0.036

ABG: arterial blood gases; AHI: apnea-hypopnea index; BDI: Beck Depression Inventory; BP: bodily pain; CVA/TIA: Cerebro-Vascular Accident/Transient Ischemic Attack; ESS: Epworth Sleepiness Scale; GH: general health; MH: mental health; NREM: Non-rapid eye movement; ODI: oxygen desaturation index; PF: physical functioning; PSG: polysomnography; RE: role emotional; REM: rapid eye movement; RP: role physical; SF: social functioning; SF-36: Short-Form-36; SpO_2_: resting room air pulse oximetry;SWS: Slow-wave sleep; TST: total sleep time; TST90: sleep time with oxygen saturation < 90%; VT: vitality.

## Data Availability

The data presented in this study are available on request from the corresponding author. The data are not publicly available due to privacy restrictions.
